# Novel Visual Sensor Coverage and Deployment in Time Aware PTZ Wireless Visual Sensor Networks

**DOI:** 10.3390/s17010064

**Published:** 2016-12-30

**Authors:** Florence G. H. Yap, Hong-Hsu Yen

**Affiliations:** 1 Center for General Education, Chang Gung University, Taoyuan 333, Taiwan; florence.ghyap@gmail.com; 2 Department of Information Management, Shih Hsin University, Taipei 116, Taiwan

**Keywords:** PTZ visual sensor, PTZ time constraint, visual sensor deployment, wireless visual sensor networks

## Abstract

In this paper, we consider the visual sensor deployment algorithm in Pan-Tilt-Zoom (PTZ) Wireless Visual Sensor Networks (WVSNs). With PTZ capability, a sensor’s visual coverage can be extended to reduce the number of visual sensors that need to be deployed. The coverage zone of a visual sensor in PTZ WVSN is composed of two regions, a Direct Coverage Region (DCR) and a PTZ Coverage Region (PTZCR). In the PTZCR, a visual sensor needs a mechanical pan-tilt-zoom operation to cover an object. This mechanical operation can take seconds, so the sensor might not be able to adjust the camera in time to capture the visual data. In this paper, for the first time, we study this PTZ time-aware PTZ WVSN deployment problem. We formulate this PTZ time-aware PTZ WVSN deployment problem as an optimization problem where the objective is to minimize the total visual sensor deployment cost so that each area is either covered in the DCR or in the PTZCR while considering the PTZ time constraint. The proposed Time Aware Coverage Zone (TACZ) model successfully captures the PTZ visual sensor coverage in terms of camera focal range, angle span zone coverage and camera PTZ time. Then a novel heuristic, called Time Aware Deployment with PTZ camera (TADPTZ) algorithm, is proposed to solve the problem. From our computational experiments, we found out that TACZ model outperforms the existing *M* coverage model under all network scenarios. In addition, as compared to the optimal solutions, the TACZ model is scalable and adaptable to the different PTZ time requirements when deploying large PTZ WVSNs.

## 1. Introduction

The Wireless Visual Sensor Network (WVSN) is a special type of Wireless Sensor Network (WSN) where the sensor node is equipped with the camera to capture visual data (e.g., images or video) [1]. The first smart camera idea equipped with a high-performance onboard computing and communication infrastructure, combining video sensing, processing, and communications in a single device appeared in [2,3]. By equipping cameras on a sensor, WVSNs enable much richer applications such as security surveillance, wildlife observation, and in-house baby care. As compared to WSNs, the sensor coverage in a WVSN is totally different due to the camera’s physical constraints. In a WSN, a sensor can acquire the scalar data (e.g., temperature) of the nodes as long as the nodes are within the sensing range of the sensor. However, in a WVSN, two camera-related physical requirements must be considered so as to successfully capture the visual data of the object.

The first requirement is the focal range, which indicates the farthest distance to capture objects in a scene with acceptable visual data quality. The second requirement is the span angle of the camera. This indicates the angle range that a camera lens can capture an image, and the angle range is usually less than 180°. These two requirements define the Field of View (FoV) coverage of the visual sensor in WVSNs.

**Figure 1:**
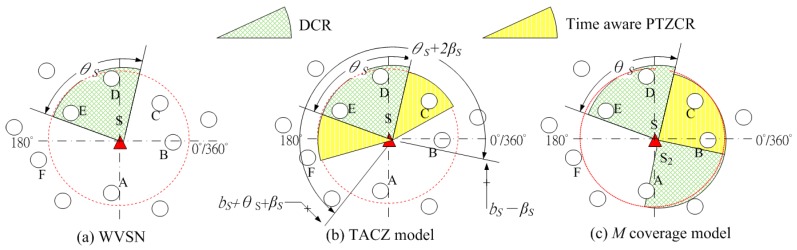
WVSN vs. PTZ WVSN in TACZ model and *M* coverage model.

Nowadays, new Pan-Tilt-Zoom (PTZ)-capable security cameras are available in the market where the camera can pan horizontally, tilt vertically and zoom in/out to extend its FoV coverage as compared to existing WVSNs. This kind of PTZ-capable WVSN is abbreviated as PTZ WVSN. Because of the new PTZ capability, the coverage model and deployment method of a PTZ WVSN is different from that of a WVSN. In Figure 1, we illustrate an example to show the coverage differences between a WVSN and a PTZ WVSN.

In Figure 1, let *b_S_* be the starting angle of the angle span zone of sensor *S*; *θ_S_* be the span angle of sensor *S*; *β_S_* be the PTZ angle of sensor *S*. In Figure 1a, node *S* is the visual sensor and the red dashed circle indicates the focal range of the sensor *S*. Then, sensor *S* can capture the events in nodes *A*, *B*, *C*, *D* and *E* with acceptable visual data quality. However, due to the span angle constraint, sensor *S* can only capture the visual data at nodes *D* and *E*. Hence, only nodes *D* and *E* are within the FoV coverage of sensor *S*. Note that in the case of WSN, the red dashed circle indicates the sensing range of the sensor *S*. In this case, sensor *S* can sense and capture the data in nodes *A*, *B*, *C*, *D* and *E*.

We define the FoV coverage area as the Direct Coverage Region (DCR) and PTZ Coverage Region (PTZCR). PTZCR is the extended FoV coverage by PTZ camera on the sensor. In Figure 1b, we illustrate an example where the PTZ camera equipped on sensor *S* can horizontally pan *β_S_* degrees and span angle of the DCR is *θ_S_* degrees. In this case, besides nodes *D* and *E*, nodes *B* and *C* are also within the FoV coverage of sensor node *S* when node *S* pans.

Basically, in a PTZ WVSN, nodes covered in PTZCR is different from the nodes covered in DCR. This is because a visual sensor needs to adjust its camera to get the visual information of the nodes covered in the PTZCR. This PTZ adjustment is a mechanical operation that takes the camera seconds or even minutes to pan, tilt or zoom an object in the PTZCR. For applications such as real-time security surveillance, the time for camera PTZ operation is too long to capture the image of the intruder. Hence, even though the PTZ camera could enlarge the FoV coverage in WVSN, the coverage region in PTZCR is different from the DCR due to the PTZ time. Intuitively, by introducing the PTZ time constraint, the PTZCR will grow or shrink in loose or stringent PTZ time requirement.

We call this PTZCR that will grow or shrink according to the PTZ time requirement as the “Time Aware PTZCR”. For example, in Figure 1b, even though node *B* is covered in the PTZCR, but it is not covered in the time aware PTZCR so that visual sensor *S* cannot capture the visual data of node *B* in time with PTZ time constraint. Next, it raises an interesting research question. What is the cost efficient solution of deploying the PTZ WVSN in considering the PTZ time constraint?

It is very important to take the requirements of network operation applications into consideration before deploying a network. In PTZ wireless visual sensor network, by leveraging on the PTZ capability, one of the most important application is real-time object tracking in security surveillance [4,5,6]. As video framing rate, 10 or 25 frames per second is mostly adopted. This makes the inter-frame spacing time be on a scale of milliseconds. For example, the inter-frame spacing time is 100 milliseconds for a framing rate at 10 frames per second. In real-time tracking applications, the processing time requirement for the object detection algorithm and camera calibration mechanism [7] is on the scale of milliseconds so as to identify the difference between frames in time. In other words, the PTZ time in seconds is not acceptable for real-time object tracking applications. In this paper, for the first time, we propose a novel PTZ WVSN deployment scheme to consider the tradeoff between deployment cost and network coverage so as to satisfy the visual data capture time constraint in network operation.

In [8], Rahimi et al. developed a small camera sensor device called Cyclops that can perform local image capture and analysis. Cyclops shows that WVSN is a more promising solution in security surveillance than traditional approaches. Most of the existing research on the sensor coverage and deployment problem in WVSN by using the camera’s span angle coverage [9,10,11,12,13,14]. Besides span angle coverage, the sensing range coverage should also be considered. In [15], Yen considered a cost-efficient WVSN deployment algorithm in considering the angle coverage and sensing range coverage simultaneously. In [16], Yap et al. extended this coverage model to consider the occlusion from the obstacles where the FoV blocking probability model is proposed for fixed and moving obstacles. In [17], Gonzalez-Barbosa et al. presented camera coverage model that addresses the angle span zone and focal range in mixed directional and omnidirectional camera network.

Some general models are proposed to consider multiple camera attributes (e.g., focal distance, angle, resolution) by using problem simplifying techniques. In [18], Erdem et al. proposed a camera placement and coverage model based on the canonical 0-1 optimization model to simplify the problem complexity. In [19], Angella et al. presented the idea of translating the set of camera characteristics into the distance to simplify computational complexity. They also presented the hardware acceleration implementation to speed up the computational time. Even though these techniques can simplify the problem complexity, but they also sacrifice the feasibility to real camera networks.

The first placement problem in PTZ WVSNs appeared in [20]. Fusco et al. proved that this placement problem is a NP-complete problem and proposed an approximation algorithm to tackle this problem. In [21], Sreedevi et al. considered sensor coverage in PTZ WVSNs. They propose a probability space model to tackle the coverage problem. The basic idea of the probability space model is that the probability of the space at the center of FoV being covered is more as compared to the other parts of the area. In [22], Konda et al. developed a PTZ camera auto configuration scheme to maximize the coverage model and visual quality in indoor environments. Interestingly, they consider the illumination of the light source and camera distortion in quantifying the captured visual data quality. 

PTZ camera networks with extended coverage via PTZ capability are good candidates in the application of real-time object tracking. In [4], Munishwar et al. considered the automatic control mechanism of the cameras to maximize coverage of a set of targets in a PTZ WVSN. In this work, the locations of the visual sensors are given, and the primal goal is to design a cooperation mechanism between the visual sensors to control the PTZ parameters so as to cover the maximum number of objects. In [5], Esterle et al. considered object tracking application via camera zooming configuration scheme in an omnidirectional (i.e., 360 degree) visual sensor network. They identify the trade-off between high confidence in object tracking and redundancy in failure scenarios. In [6], Kang et al. proposed a background generation algorithm in considering the camera’s pan and tilt movement to detect moving objects. In [23,24], Starzyk et al. proposed a PTZ camera control and handoff mechanism to track moving objects. They introduced a concept of conditional offers during PTZ camera handoff negotiations. This conditional offers allow successful handoff to identify the right PTZ camera for object tracking. In [25], Dieber et al. considered an interesting visual sensor network (VSN) configuration problem which jointly considered network coverage and resource allocation problems in resource limited VSN. In this work, two key questions are answered: how to identify the cameras to cover the area of interest so as to minimize energy usage and maximize the network lifetime and how to identify the settings of camera’s frame rate and resolution to fulfill the quality of service requirements. The tradeoff between surveillance quality and resource utilization has been identified in the proposed multi-criteria approximation algorithm. This VSN configuration algorithm also extends to the camera with the PTZ capability. In [26] Dieber et al. applied the idea of a VSN configuration algorithm in the object tracking application.

However, in these previous works on PTZ WVSNs, the PTZ time constraint is not addressed; hence, they are not applicable to time constrained applications. The first PTZ time-aware PTZ WVSN deployment appears in [27,28]. In these two works, Yen introduced the idea of *M* coverage where the DCR is covered by one visual sensor, but the PTZCR is covered by multiple (*M*) visual sensors. The basic idea of the *M* coverage model is to increase the visual coverage probability and to decrease the visual data capture time of the moving objects in PTZCR via the cooperation of multiple camera sensors. To be more specific, when an object is covered in the PTZCR of multiple visual sensors, the possibility of at least one camera sensor being able to PTZ the camera in time to capture the visual data is higher than the PTZCR coverage of only one sensor. For example, in Figure 1c, we have two deployed visual sensors, *S*_1_ and *S*_2_. In this case, the right PTZCR of sensor *S*_1_ overlapped with the left PTZCR of sensor *S*_2_ so nodes *B* and *C* are considered to be covered in the PTZCR of two visual sensors. Even though *M* coverage can reduce the PTZ time via multiple visual sensors but this *M* coverage model cannot adapt to different PTZ time constraint. As shown in the computational experiments, the *M* coverage model will deploy too many visual sensors under loose time constraint conditions and it cannot locate feasible solutions under stringent time constraints.

In this paper, we propose a time-aware PTZ optimization model called Time Aware Coverage Zone (abbreviated as TACZ) and a novel heuristic, Time Aware Deployment with PTZ camera (abbreviated as the TADPTZ algorithm) to tackle PTZ WVSN deployment problems in considering the focal range, angle span zone and PTZ time. In the following, we summarize the contributions of this paper:

- Novel time aware PTZ WVSN sensor coverage model: We propose a rigorous mathematical model, TACZ, to capture sensor coverage in the DCR and PTZCR with three criteria (focal range, camera angle span zone, and camera PTZ time).
- Novel time aware PTZ WVSN sensor deployment model and algorithm: PTZ time aware PTZ WVSN deployment is formulated as an optimization problem where the objective is to identify a minimum sensor deployment cost so as to ensure each node being either covered in the DCR or covered in the time-aware PTZCR of a deployed sensor. Then the optimization-based algorithm is proposed to tackle this problem.
- PTZ WVSN deployment solution with good scalability to the PTZ time requirement: The proposed model and algorithm is scalable and adaptable to the PTZ time constraint. In addition, the proposed algorithm outperforms the existing *M* coverage model under all network setting conditions.

## 2. Sensor Coverage Model in a PTZ WVSN

Basically, there are two types of camera coverage model. The first type of camera coverage model considers the camera’s planar coverage where the camera’s view is horizontal [25,29]. A typical example would be security surveillance with camera mounted on the ceiling beside the door. The second type of camera coverage model considers the camera’s bird view coverage where the camera’s view is vertical [30]. A typical example would be a quadcopter drone equipped with a camera. In the first type of camera’s planar coverage model, the coverage of the camera is modeled as a 2D sector where the camera’s focal range is the radius of the sector and the camera’s span angle is the central angle of the sector. In the second type of camera bird’s eye view coverage model, the coverage of the camera is modeled as the 2D circle from the 3D sphere where the radius of the circle is the camera’s focal range times sin (*θ*/2) which *θ* is the camera’s span angle. In this paper, we adopt the first type of camera planar coverage model where the camera’s coverage zone is modeled as a 2D sector with the camera’s focal range being the radius of the sector and the camera’s span angle being the central angle of the sector.

The camera angle span zone requirement comes from the view span angle of the camera. With the PTZ capability, the camera’s view angle can be extended. Figure 1b shows the new extended coverage area, PTZCR.

Let *b_S_* be the starting angle of the angle span zone of sensor *S*; *θ_S_* be the span angle of sensor *S*; *β_S_* be the PTZ angle of sensor *S* and *a_iS_* be the relative angle of node *i* to sensor *S*. In Figure 1b, a node *i* is within the DCR and PTZCR of a visual sensor *S* if:

DCR coverage:

(1) $$b_{S}\leq a_{iS}\leq b_{S}+\theta_{S}$$

PTZCR coverage:

(2) $$b_{S}-\beta_{S}\leq a_{iS}\leq b_{S}$$

or:

(3) $$b_{S}+\theta_{S}\leq a_{iS}\leq b_{S}+\theta_{S}+\beta_{S}$$

However, Equations (1)–(3) do not consider the cases when the angle span zone covers 0/2π. Note that in the polar grid, when the angle is larger than 2π or less than 0, it should be normalized between 0 to 2π. Based on the results of our earlier work in [27,28], the angle span zone is modified as Equations (4)–(9) for angle normalization at sensor *j*.

DCR angle coverage:

(4) $$b_{j}\leq a_{ij}\leq b_{j}+\theta_{j}\;\forall i,j\in L$$

or:

(5) $$a_{ij}\leq b_{j}+\theta_{j}-2\pi \;\forall i\in L,j\in \left\{k|b_{k}+\theta_{k}>2\pi ,\;k\in L\right\}$$

PTZCR angle coverage:

(6) $$b_{j}-\beta_{j}\leq a_{ij}\leq b_{j}\;\forall i,j\in L$$

or:

(7) $$b_{j}+\theta_{j}\leq a_{ij}\leq b_{j}+\theta_{j}+\beta_{j}\;\forall i,j\in L$$

or:

(8) $$a_{ij}\leq b_{j}+\theta_{j}+\beta_{j}-2\pi \;\forall i\in L,j\in \left\{k|b_{k}+\theta_{k}+\beta_{k}>2\pi ,\;k\in L\right\}$$

or:

(9) $$b_{j}-\beta_{j}+2\pi \leq a_{ij}\;\forall i\in L,j\in \left\{k|b_{k}-\beta_{k}<0,\;k\in L\right\}$$

As we state in the Section 1, the FoV coverage in PTZCR is different from the FoV coverage in DCR because the visual sensor need to mechanically adjust the camera to capture the visual data in the PTZCR. In the next section, we deal with the PTZ time directly. Hence, if a node *i* is covered in the PTZCR of a visual sensor *j*, then the PTZ time for sensor *j* must be within the maximum time threshold *T*. Note that Equations (4)–(9) specify the two dimensional (2D) angle span zone for DCR and PTZCR. In the next section, we will extend 2D angle span zone to three dimensional (3D) angle span zone for DCR and PTZCR.

## 3. Time-Aware Coverage Zone (TACZ) Model

In Section 2, we only address the focal range and angle zone coverage of DCR and PTZCR in a PTZ WVSN. In this Section, we propose the TACZ model to capture the minimum cost sensor deployment problem in PTZ WVSN with time constraints. In the TACZ model, the deployment area of a WVSN is modeled as a graph and every possible location to deploy a sensor or to be covered by the deployed sensor is denoted as the node. For a node to be covered by the deployed sensor, there are three requirements (i.e., camera focal range coverage, angle zone coverage and PTZ time) that are needed to be satisfied. The focal range means that distance between the node and the sensor node should not be larger than the focal range of the camera equipped on the sensor. The angle zone coverage includes the DCR and PTZCR angle coverage as indicated at Section 2. The PTZ time constraint requires that if the node is covered in the PTZCR of a visual sensor, then the PTZ time must be within the maximum allowable time threshold, *T*. The notations used in the formulation are listed in Table 1:

The TACZ mathematical model formulation is proposed as follows:

Problem (P):

(10) $$Z_{P}=\min\sum_{j\in L}\Psi_{j}\left(D_{j}\right)$$

Subject to:

(11) $$1\leq \sum_{j\in L}v_{ij}^{1}+\sum_{j\in L}v_{ij}^{2}\;\forall i\in L$$

(12) $$v_{ij}^{1}\leq D_{j}\;\forall i,j\in L$$

(13) $$v_{ij}^{2}\leq D_{j}\;\forall i,j\in L$$

(14) $$d_{ij}\times v_{ij}^{k}\leq r_{j}\;\forall i,j\in L,k=1,\;2$$

(15) $$z_{ij}^{1}\leq \max\left\{\frac{a_{ij}-b_{j}+\varepsilon }{\varepsilon },\;0\right\}\;\forall i,j\in L$$

(16) $$z_{ij}^{1}\leq \max\left\{\frac{b_{j}+\theta_{j}-a_{ij}+\varepsilon }{\varepsilon },\;0\right\}\;\forall i,j\in L$$

(17) $$z_{ij}^{2}\leq \max\left\{\frac{b_{j}+\theta_{j}-a_{ij}-2\pi +\varepsilon }{\varepsilon },\;0\right\}\;\forall i\in L,j\in \left\{k|b_{k}+\theta_{k}>2\pi ,\;k\in L\right\}$$

(18) $$v_{ij}^{1}\leq z_{ij}^{1}+z_{ij}^{2}\;\forall i,j\in L$$

(19) $$y_{ij}^{1}\leq \max\left\{\frac{a_{ij}-b_{j}+\beta +\varepsilon }{\varepsilon },\;0\right\}\;\forall i,j\in L$$

(20) $$y_{ij}^{1}\leq \max\left\{\frac{b_{j}-a_{ij}+\varepsilon }{\varepsilon },\;0\right\}\;\forall i,j\in L$$

(21) $$y_{ij}^{2}\leq \max\left\{\frac{b_{j}+\theta_{j}+\beta -a_{ij}+\varepsilon }{\varepsilon },\;0\right\}\;\forall i,j\in L$$

(22) $$y_{ij}^{2}\leq \max\left\{\frac{a_{ij}-b_{j}-\theta_{j}+\varepsilon }{\varepsilon },\;0\right\}\;\forall i,j\in L$$

(23) $$y_{ij}^{3}\leq \max\left\{\frac{b_{j}+\theta_{j}+\beta_{j}-a_{ij}-2\pi +\varepsilon }{\varepsilon },\;0\right\}\;\forall i\in L,j\in \left\{k|b_{k}+\theta_{k}+\beta_{k}>2\pi ,\;k\in L\right\}$$

(24) $$y_{ij}^{4}\leq \max\left\{\frac{a_{ij}-b_{j}+\beta_{j}-2\pi +\varepsilon }{\varepsilon },\;0\right\}\;\forall i\in L,j\in \left\{k|b_{k}-\beta_{k}<0,\;k\in L\right\}$$

(25) $$v_{ij}^{2}\leq y_{ij}^{1}+y_{ij}^{2}+y_{ij}^{3}+y_{ij}^{4}\;\forall i,j\in L$$

(26) $$v_{ij}^{2}\times \Gamma_{j}\left(a_{ij}\right)\leq T\;\forall i,j\in L$$

(27) $$z_{ij}^{k}=0\;\mathrm{or}\;1\;\forall i,j\in L,k=1,\;2$$

(28) $$y_{ij}^{k}=0\;\mathrm{or}\;1\;\forall i,j\in L\;k=1,2,3,4$$

(29) $$v_{ij}^{k}=0\;\mathrm{or}\;1\;\forall i,j\in L,k=1,\;2$$

(30) $$D_{j}=0\;\mathrm{or}\;1\;\forall j\in L$$

The objective function is to minimize the total deployment cost. Constraint (11) enforces that every node is at least covered within the DCR of one deployed sensor or within the PTZCR of the deployed sensor. Constraint (12) and (13) enforce that sensor at location *j* must be deployed first before it can sense and capture the visual data of the DCR and PTZCR. Constraint (14) is the camera focal range requirement, which specifies that if location *j* is within the range, then the distance between location *i* and *j* must be less than the focal range of sensor *i*.

In considering the Constraints (15) and (16), when Equation (4) is not satisfied, the decision variable $z_{ij}^{1}$ will be 0. On the other hand, $z_{ij}^{1}$ could be 1 when Equation (4) is satisfied. Similarly, the same idea also applies to Constraint (17) and Equation (5). At Constraint (18), if any $z_{ij}^{1}$ = 1 or $z_{ij}^{2}$ = 1, then node *i* is covered within the DCR of sensor *j* (i.e., $v_{ij}^{1}=1$ ). In other words, Constraints (15)–(18) successfully capture the idea of DCR angle coverage defined in Equations (4) and (5).

Next we deal with the angle coverage constraints in PTZCR. Constraints (19) and (20) to enforce $y_{ij}^{1}$ could be 1 when Equation (6) is satisfied. Constraints (21) and (22) to enforce $y_{ij}^{2}$ could be 1 when Equation (7) is satisfied; Constraints (23) to enforce $y_{ij}^{3}$ could be 1 when Equation (8) is satisfied; Constraints (24) to enforce $y_{ij}^{4}$ could be 1 when Equation (9) is satisfied. At Constraint (25), it specifies that if any $y_{ij}^{k}=1$ ∀k=1, 2, 3, 4 , then node *i* is covered within the angle coverage of the PTZCR of sensor *j* (i.e., $v_{ij}^{2}=1$ ). Hence, Constraints (19)–(25) successfully capture the idea of PTZCR coverage defined in Equations (6)–(9).

Constraint (26) specifies the camera adjustment time constraint in PTZCR. On the left hand side of Constraint (26), by multiplying the camera adjustment time $\Gamma_{j}\left(a_{ij}\right)$ with the decision variable $v_{ij}^{2}$ , the camera adjustment time constraint is only valid in the PTZCR. Hence, in Constraint (26), to capture a node in PTZCR, the camera adjustment time must not exceed the maximum allowable threshold *T*. Constraints (27)–(30) specify the feasible region of the decision variables.

Note that by enforcing Constraint (26), the PTZCR might shrink under stringent PTZ time constraints (i.e., *T* is very small). For example, in Figure 1b, the angle span zone in DCR and PTZCR of visual sensor *S* is $\left(\theta_{S}+2\beta_{S}\right)$ . In this case, originally, the PTZCR of visual sensor *S* covers nodes *B* and *C*. However, due to the stringent PTZ time constraint, the time aware PTZCR is the yellow region that can only cover node *C*. So in this case, the time aware PTZCR is smaller than the PTZCR. When in the case of loose PTZ time constraint (i.e., *T* is very big), then Constraint (26) is not binding so that the time aware PTZCR is the same as the PTZCR. In other words, by enforcing Constraint (26), the coverage zone of a deployed sensor in problem (P) is the union of DCR and time aware PTZCR. In the following, we prove that there exists an optimal solution of the TACZ model in Problem (P).

Corollary: There exists an optimal solution of the TACZ model in Problem (P).

Proof: Proof by contradiction.

Assume that ϑ is an optimal solution of the TACZ model and ϑ is not a feasible solution in Problem (P). Since ϑ is an optimal solution of the TACZ model, then the three requirements (i.e., camera focal range coverage, angle zone coverage and PTZ time) for placing the wireless visual sensor nodes should be satisfied. To satisfy the first focal range requirement, it means that distance of the node and the deployed sensor to cover this node must be not larger than the focal range of this deployed sensor (i.e., Constraint (14) should be satisfied). To satisfy the second angle zone coverage requirement, it means that the DCR angle coverage constraint (i.e., Constraints (15)–(18)) and the PTZCR angle coverage constraint (i.e., Constraints (19)–(25)) should be satisfied. To satisfy the third PTZ time constraint, it means that the camera adjustment time constraint in Constraint (26) should be satisfied. Besides these three requirements, for ϑ to be an optimal solution, it ensures that every interested location will be covered either by the DCR or the PTZCR of the deployed sensor nodes in ϑ. Hence, the Constraints (11)–(13) will also be satisfied. 

Since the deployed sensors in ϑ satisfy all the Constraints (11)–(30), ϑ is a feasible solution in Problem (P) which violate the assumption that ϑ is not a feasible solution in Problem (P). Then there exist an optimal solution in the TACZ model in Problem (P).

To summarize, in TACZ mathematical model, we successfully capture the PTZ WVSN deployment problem in considering the three sets of constraints. The first one is camera focal range constraint (Constraint (14)). The second one is the angle span coverage which includes the DCR angle coverage (Constraints (15)–(18)) and PTZCR angle coverage (Constraints (19)–(25)). The third one is the PTZ time constraint (Constraint (26)).

Note that in this proposed TACZ model, even though we consider the 2D coverage in terms of the Pan operation, the TACZ model can be easily extended to incorporate the 3D coverage problem by introducing another set of Tilt angle variables. Hence, we have:

DCR coverage in Tilt:

$$\bar{b_{S}}\leq \bar{a_{iS}}\leq \bar{b_{S}}+\bar{\theta }$$

PTZCR coverage in Tilt:

$$\bar{b_{S}}-\bar{\beta }\leq \bar{a_{iS}}\leq \bar{b_{S}}$$

or:

$$\bar{b_{S}}+\bar{\theta }\leq \bar{a_{iS}}\leq \bar{b_{S}}+\bar{\theta }+\bar{\beta }$$

where the variable with the bar indicates the angle with the Tilt operation, which is the same as the variable without the bar in Pan angle (i.e., $\bar{a_{iS}}$ is the same as $a_{iS}$ but in Tilt angle).

Then in the 3D TACZ coverage, nine Tilt angle constraints (i.e., Constraints (15-1)–(17-1) and Constraints (19-1)–(24-1)) should be added. For example, Constraint (15-1) is:

$$z_{ij}^{1}\leq \max\left\{\frac{\bar{a_{ij}}-\bar{b_{j}}+\varepsilon }{\varepsilon },\;0\right\}\;\forall i,j\in L$$

Besides these nine Tilt angle constraints, the camera PTZ time should add the time in tilting. Then Constraint (26) becomes:

$$v_{ij}^{2}\times \left(\Gamma_{j}\left(a_{ij}\right)+\Gamma_{j}\left(\bar{a_{ij}}\right)\right)\leq T\;\forall i,j\in L$$

Basically, new constraints associated with the Tilt operation restricts the feasible solution space of the problem (P). However, these new constraints are all linear so that they do not change the mathematical structure of problem (P). In other words, problem (P) can be easily extended to consider the 3D coverage. It is worthwhile to mention that in Constraint (26), the PTZ time only considers the time on panning and tilting but without addressing the time on zooming. This is because the zooming operation is usually manually operated by human, which is not available in the large scale PTZ WVSN. Next, we show the TADPTZ algorithm.

## 4. Solution Approach—TADPTZ Algorithm

PTZ WVSN deployment problem has been proven to be a NP-complete problem in [20]. We propose the novel TADPTZ algorithm to tackle this problem. Basically, by leveraging on the PTZ capability, Time Aware Coverage Zone (TACZ) is the union of DCR and camera PTZ time aware PTZCR. Let ${\Gamma_{j}}^{-1}\left(T\right)$ be the maximal adjustment angle on camera sensor *j* with maximum allowable adjustment time threshold *T*. In considering the loose PTZ time constraint, ${\Gamma_{j}}^{-1}\left(T\right)$ < $\beta_{j}$ , then the new PTZ angle of sensor *j* is ${\Gamma_{j}}^{-1}\left(T\right)$ . In loose PTZ time constraint, ${\Gamma_{j}}^{-1}\left(T\right)$ ≥ $\beta_{j}$ , then the PTZ angle of sensor *j* is $\beta_{j}$ . We introduce variable $\zeta_{j}$ to consider these two conditions.

Let $\zeta_{j}$ be the camera adjustment time aware PTZ angle of sensor *j*, then:

(31) $$\zeta_{j}={\Gamma_{j}}^{-1}\left(T\right)\;\forall j\in \left\{i|i\in L,\;{\Gamma_{i}}^{-1}(T)<\beta_{i}\right\}$$

(32) $$\zeta_{j}=\beta_{j}\;\forall j\in \left\{i|i\in L,\;{\Gamma_{i}}^{-1}(T)\geq \beta_{i}\right\}$$

Then without considering the angle normalization, for node *i* to be covered in the TACZ of sensor *j*, the TACZ coverage requirement is:

(33) $$b_{j}-\zeta_{j}\leq a_{ij}\leq b_{j}+\theta +\zeta_{j}\;\forall i,j\in L$$

With considering the angle normalization between 0 to 2π , the TACZ angle coverage requirement is revised as:

(34) $$b_{j}-\zeta_{j}\leq a_{ij}\leq b_{j}+\theta +\zeta_{j}\;\forall i,j\in L$$

or:

(35) $$a_{ij}\leq b_{j}+\theta_{j}+\zeta_{j}-2\pi \;\forall i\in L,j\in \left\{k|b_{k}+\theta_{k}+\zeta_{k}>2\pi ,\;k\in L\right\}$$

or:

(36) $$b_{j}-\zeta_{j}+2\pi \leq a_{ij}\;\forall i\in L,j\in \left\{k|b_{k}-\zeta_{k}<0,\;k\in L\right\}$$

Note that besides the angle coverage constraint, another focal range constraint (i.e., Constraint (14)) must also be satisfied in TACZ. We propose a heuristic algorithm called the TADPTZ algorithm to tackle the TACZ model. The basic idea of the TADPTZ algorithm is to deploy a sensor that can cover the largest number of uncovered nodes. This process iterates until all the nodes (i.e., locations) in the network are covered in the TACZ of the deployed sensors. In the iterative process, every time a new sensor is deployed, it will cover some uncovered nodes and it will also cover the covered nodes by the sensors deployed in the earlier stages. In some of the cases, this offers the possibility to reduce the deployment cost. That is, when a new visual sensor is deployed, if the TACZ coverage area of the sensor (say sensor *q*) deployed at the earlier stage could be fully covered by other deployed sensors, then sensor *q* could be removed to reduce the deployment cost. We call this the TACZ recheck procedure.

Note that in the TACZ recheck procedure, every time a new visual sensor is deployed, the other deployed sensors will check their TACZ to see if it is fully covered by other deployed sensors. To speed-up the checking process, a set based theory is proposed as follows. Let *TACZ_i_* be the set of covered nodes by sensor *i*, Ω denotes the set of covered nodes in the network, and Ω_new_ is the new set of covered nodes after including the new deployed sensor (say sensor *i*). Then we have:

(37) $$\Omega_{\mathrm{new}}=\Omega \cup TACZ_{i}$$

For every sensor deployed in the earlier stages, if its *TACZ_j_* is a partial set of Ω_new_, then remove this sensor (say sensor *j*) and adjust the set Ω_new_. In other words:

if:

(38) $$TACZ_{j}\subseteq \Omega_{\mathrm{new}}$$

then:

(39) $$\Omega_{\mathrm{new}}=\Omega_{\mathrm{new}}-TACZ_{j}$$

In [16], we propose a link list data structure to reduce the computational complexity of computing the DCR coverage in WVSN. This link list data structure could also be used to speed-up the set operation in Equations (37)–(39). In the following, we show the complete TADPTZ algorithm (Algorithm 1):
**Algorithm 1. TADPTZ Algorithm** **Begin** **Initialize** the value of all the decision variables to be zero; **Determine**
*ζ_j_* for each possible sensor *j* based on the Equations (31) and (32); **Calculate** the TACZ for each possible sensor based on the focal range Constraint (14) and angle coverage constraint in Equations (34)–(36);//Step 0 
α=1
; **While** (
α≠0
) **Begin** **Calculating** the number of covered nodes that has not been covered yet for every possible location *j* to deploy a sensor; //Step 1 **Identifying** location *i* that can cover the largest number of uncovered nodes; //Step 2 **Deploy** sensor *i* and update the set of covered nodes Ω based on Equation (37); //Step 3 **Perform** TACZ recheck procedure to remove the sensors deployed at earlier stages based on Equations (38) and (39); //Step 4 **Updating** the corresponding decision variables; **If** Constraint (11) is satisfied  
α=0
; **End//while** **End**

In the beginning of the TADPTZ algorithm, Step 0, determines the TACZ for each possible deployed sensor where the coverage zone of each sensor is the union of the DCR and time aware PTZCR as defined in Equations (34)–(36). The computational complexity of the TADPTZ algorithm is bounded at the “While” loop. In the worst case, every possible location will deploy a visual sensor to meet the DCR and time aware PTZCR coverage constraint. It indicates that the “While” loop have to loop for |L| times in the worst case.

In step 1 and step 2 of TADPTZ algorithm, it is to identify the sensor that can cover the largest number of uncovered nodes. The computational complexity is $O\left({\left|L\right|}^{2}\right)$ in step 1 and step 2. In step 3, it is to deploy a sensor and update the set of covered nodes Ω after deploying this sensor. The computational complexity is O(|L|) in step 3. In step 4, it is to identify and remove the sensor having TACZ as a partial set of Ω (i.e., the covered nodes could be fully covered by the other sensors). We adopt the link list data structure in our earlier work [16] to record the list of visual sensors to cover a node. Let *ξ* denotes the maximum number of visual sensors that cover this node; then to remove a deployed sensor, we have to make sure there is at least one visual sensor in the link list for every node after removing this visual sensor. Thus, the computational complexity for the step 4 is $O\left(\xi^{2}\times \left|L\right|\right)$ . Because ξ<<|L| , then the computational complexity inside the “While” loop is $O\left(\xi^{2}\times \left|L\right|+{\left|L\right|}^{2}\right)$ . Because the “While” loop is at most to loop |L| times, the computational complexity for the TADPTZ algorithm is $O\left(\xi^{2}\times {\left|L\right|}^{2}+{\left|L\right|}^{3}\right)$ .

## 5. Computational Experiments

We evaluate the performance of our TACZ model via computational experiments over a randomly generated network in the 250 × 250 area. In the set of possible nodes *L*, 1000 nodes are randomly placed in the network. We compare the solution quality of our proposed TACZ model with the *M* coverage model proposed in [27,28]. 

In the first set of experiments, we compare number of deployed sensors (denoted as *N*) in different network settings, which include focal range (i.e., *r*), DCR angle (i.e., *θ*), PTZ angle (i.e., *β*) in loose camera PTZ time (i.e., *T*) requirement in loose PTZ time constraint. Note that $\zeta_{j}$ is the camera adjustment time aware PTZ angle of sensor *j*, which is defined in Equations (31) and (32). In loose PTZ time requirement, Constraint (26) is always satisfied for the nodes covered in the PTZCR, then according to Equation (31), *ζ_j_* = *β_j_*, ∀j∈L . In the second set of experiments, we compare the solution quality at stringent PTZ time requirement, then according to Equation (32), $\zeta_{j}$ = ${\Gamma_{j}}^{-1}\left(T\right)$ , ∀j∈L . In these two sets of computational experiments, the results of *M*2 (i.e., the PTZCR is covered by two visual sensors) and *M*3 (i.e., the PTZCR is covered by three visual sensors) are compared with our proposed TACZ model.

**Figure 2:**
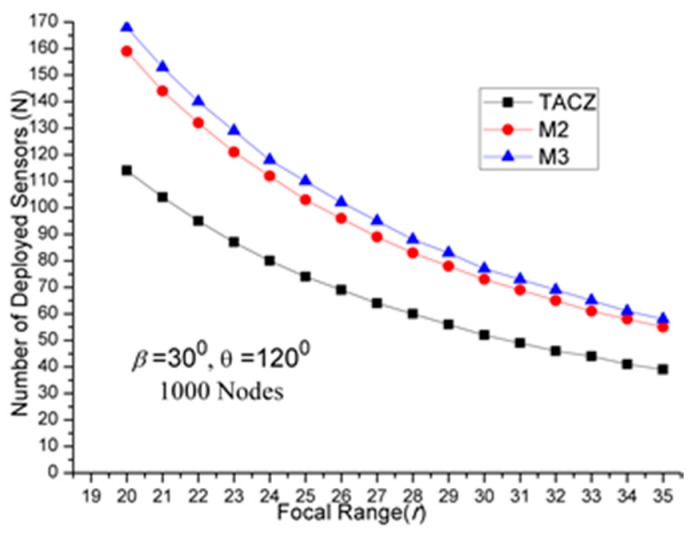
*N* w.r.t. *r* in loose PTZ time constraint.

**Figure 3:**
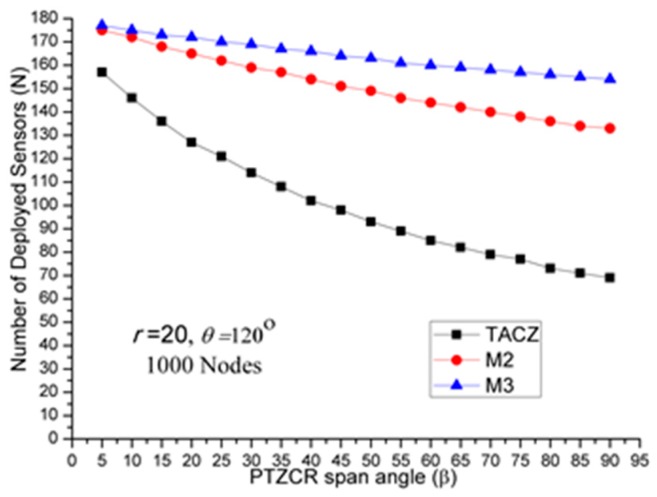
*N* w.r.t. *β* in loose PTZ time constraint.

**Figure 4:**
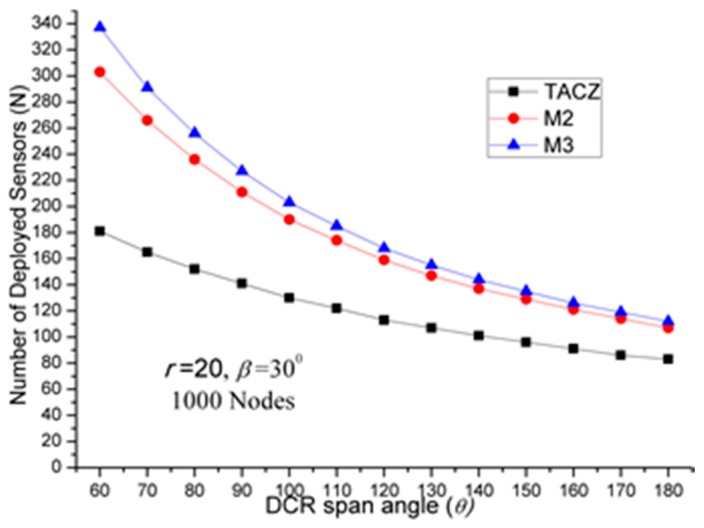
*N* w.r.t. *θ* in loose PTZ time constraint.

The results of first set of computational experiments are shown in Figure 2, Figure 3 and Figure 4. By defining the Superiority Ratio (SR) as:

(40) SR = ((MX − TACZ)/TACZ) × 100%

where MX is the number of deployed sensors for M = X coverage in [27,28] and TACZ is the number of deployed sensors (*N*) calculated by TADPTZ algorithm. In Table 2, we summarize the results. 

We have three important observations:

(a) TACZ outperforms the *M* coverage with respect to all tested focal ranges:
  In Figure 2, we observe that the TACZ outperforms the *M* coverage based algorithm under all tested focal ranges. The SR of TACZ over 2 coverage (i.e., *M2*) is about 41% and the SR of TACZ over 3 coverage (i.e., *M3*) is about 49%. This SR is almost constant regarding different values of *r*. This is because by increasing the focal range, it also increases the coverage area of DCR and PTZCR so that fewer visual sensors are needed to cover the network for both TACZ model and *M* PTZCR coverage model.
(b) TACZ outperforms the *M* coverage with respect to all tested values of *β*, especially at bigger values of *β*:
  In Figure 3, we observe that the TACZ outperforms the *M* coverage based algorithm under all tested *β*. At small *β* (i.e., *β* = 5°), the SR of TACZ over *M2* is about 11% and the SR of TACZ over *M3* is about 13%. At large *β* (i.e., *β* = 90°), the SR of TACZ over *M2* is about 93% and the SR of TACZ over *M3* is about 123%. This indicates that in loose PTZ time constraint, the advantage of TACZ model is more significant in large PTZ span angle (*β*).
(c) TACZ outperforms the *M* coverage with respect to all tested values of *β*, especially at smaller value of *θ*:
  In Figure 4, we observe that the TACZ outperforms the *M* coverage based algorithm under all tested *θ*.
  At small *θ* (i.e., *θ* = 60°), the SR of TACZ over *M2* is about 67% and the SR of TACZ over *M3* is about 86%. At large *θ* (i.e., *θ* = 180°), the SR of TACZ over *M2* is about 29% and the SR of TACZ over *M3* is about 35%. This indicates that in fixed value of *β*, by decreasing the value of *θ*, the coverage area of the PTZCR becomes more important and the SR value of TACZ over *M*2 and *M*3 becomes bigger.

In the second set of experiments, we compare the solution quality at stringent PTZ time requirement when *ζ* = 0.5*β*. That is, the time aware PTZCR is half the size of original PTZCR. In Table 3, we summarize the results. We have three important observations.

**Figure 5:**
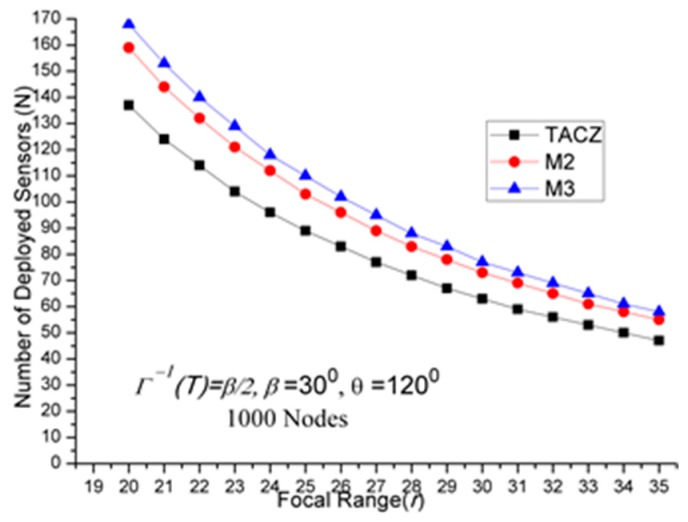
*N* w.r.t. *r* in stringent PTZ time constraint.

**Figure 6:**
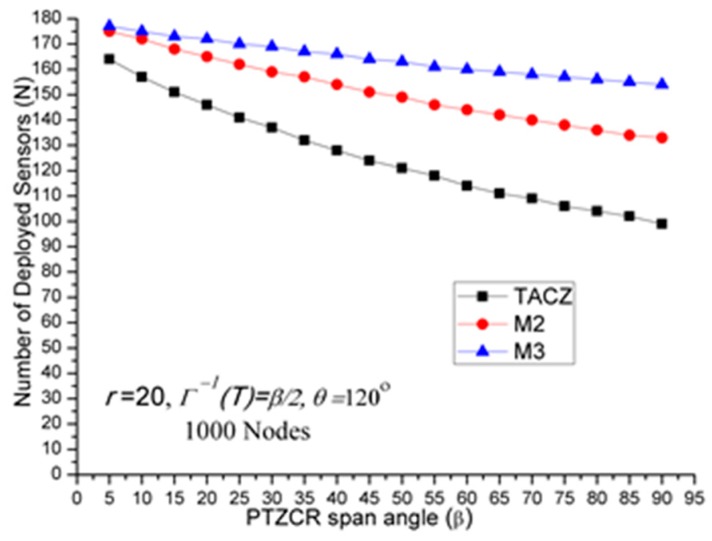
*N* w.r.t. *β* in stringent PTZ time constraint.

**Figure 7:**
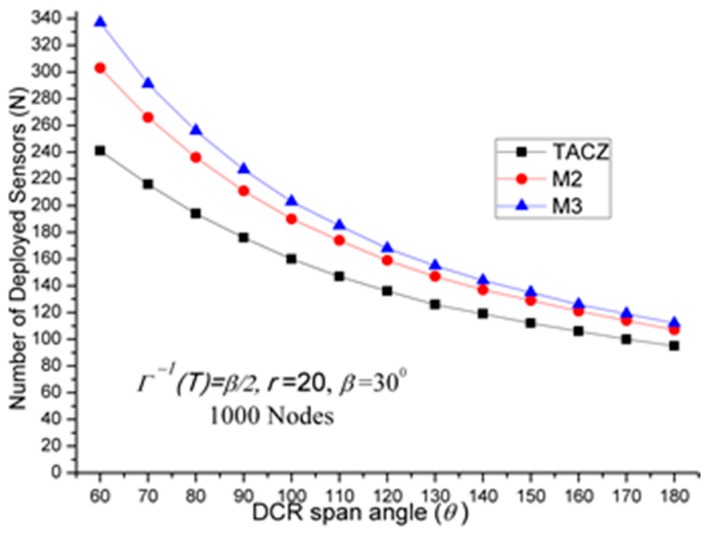
*N* w.r.t. *θ* in stringent PTZ time constraint.

(a) TACZ outperforms the *M* coverage with respect to all tested focal ranges:
  In Figure 5, we observe that the TACZ outperforms the *M* coverage based algorithm under all tested focal range.
  The SR of TACZ over M2 coverage is about 16% and the SR of TACZ over M3 coverage is about 23%. The SR is smaller than in Figure 2 because the number of deployed sensors has increased for TACZ model but remained the same for *M* coverage model. The *M* coverage model requires the PTZCR to be covered by at least *M* visual sensors. With this requirement, the camera adjustment angle to locate any node in the PTZCR is smaller than half of the *β* so that the number of deployed visual sensors remains the same for *M*2 and *M*3 coverage model.
(b) TACZ outperforms the *M* coverage with respect to all tested values of *β*, especially at bigger value of *β*:
  In Figure 6, we observe that the TACZ outperforms the *M* coverage based algorithm under all tested *β*. At small *β* (i.e., *β* = 5°), the SR of TACZ over *M2* is about 7% and the SR of TACZ over *M3* is about 8%. At large *β* (i.e., *β* = 90°), the SR of TACZ over *M2* is about 34% and the SR of TACZ over *M3* is about 56%. The SR is smaller than in Figure 3 because the number of deployed sensors increased for TACZ model but remain the same for *M* coverage model.
(c) TACZ outperforms the *M* coverage with respect to all tested values of *β*, especially at smaller value of *θ*:
  In Figure 7, we observe that the TACZ outperforms the *M* coverage based algorithm under all tested *θ*. At small *θ* (i.e., *θ* = 60°), the SR of TACZ over *M2* is about 26% and the SR of TACZ over *M3* is about 40%. At large *θ* (i.e., *θ* = 180°), the SR of TACZ over *M2* is about 13% and the SR of TACZ over *M3* is about 18%. The SR is smaller than in Figure 4 because the number of deployed sensors has increased for TACZ model but remained the same for *M* coverage model.

As compared to Table 3 to Table 2, we have one important observation. The *N* value in the TACZ model will increase as the PTZ time constraint becomes more stringent. On the other hand, the *N* value in *M* coverage model does not change as PTZ time constraint becomes more stringent when *ζ* = 0.5*β*. Then, the SR of TACZ model over *M* coverage model in Table 3 is smaller than in Table 2. So, an interesting question to be asked is: Will SR be even smaller when the PTZ time constraint gets even more stringent, say *ζ* = 0.1*β*?

In the third set of computational experiments, we answer the above question by studying the solution quality comparison with respect to PTZ span angle *β* in different PTZ time constraint. In Table 4, we summarize the results. 

**Figure 8:**
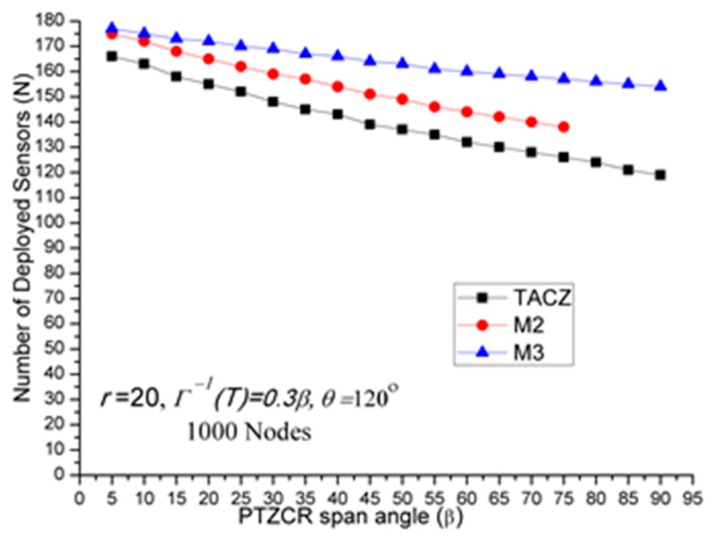
*N* w.r.t. *β* in stringent PTZ time constraint ( $\Gamma^{-1}\left(T\right)=\;0.3\beta$ ).

**Figure 9:**
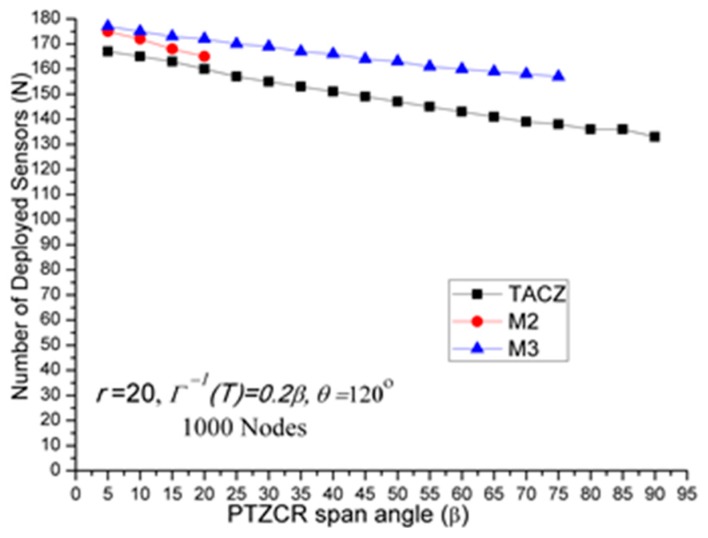
*N* w.r.t. *β* in stringent PTZ time constraint ( $\Gamma^{-1}\left(T\right)=\;0.2\beta$ ).

**Figure 10:**
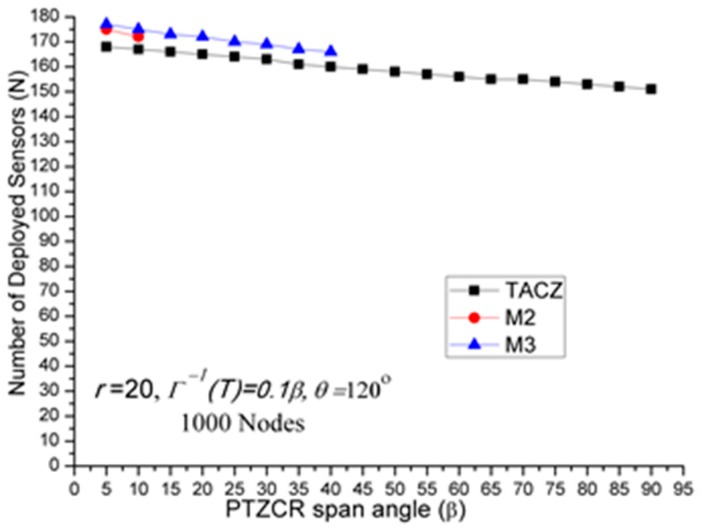
*N* w.r.t. *β* in stringent PTZ time constraint ( $\Gamma^{-1}\left(T\right)=\;0.1\beta$ ).

We have three important observations:

(a) Stringent time constraints will reduce the advantage of a large PTZ span angle (*β*) in the TACZ model:
  In Figure 3, *N* is 157 when *β* = 5° and *N* is 69 when *β* = 90°. In Figure 6, *N* is 164 when *β* = 5° and *N* is 99 when *β* = 90°. In Figure 8, *N* is 166 when *β* = 5° and *N* is 119 when *β* = 90°. In Figure 9, *N* is 167 when *β* = 5° and *N* is 133 when *β* = 90°. In Figure 10, *N* is 168 when *β* = 5° and *N* is 151 when *β* = 90°.
  This shows that in the TACZ model, under more stringent PTZ time constraint, *N* will increase more rapidly at large *β*. This is because the time aware PTZ will shrink significantly at more stringent PTZ time constraint requiring more visual sensors to cover the network. For example, in Figure 1b, the yellow time aware PTZCR zone will be even smaller when in more stringent PTZ time constraint.
(b) Stringent time constraints will not change the number of deployed sensors but will increase the probability of not finding feasible solutions in the *M* coverage model:
  Recall that *M* coverage model cannot adapt to PTZ time constraint. As summarized in Table 4, when *ζ* = 0.3*β*, *M*2 could not locate feasible solutions o*β* > 75°; when *ζ* = 0.2*β*, *M*2 could not locate feasible solutions o*β >* 20°, and *M*3 could not locate feasible solutions o*β* > 75°; when *ζ* = 0.1*β*, *M*2 could not locate feasible solutions o*β* > 10° and *M*3 could not locate feasible solutions o*β* > 40°. This implies that, under loose PTZ time constraint conditions, the *M* coverage model is too conservative, resulting in more visual sensors needing to be deployed. On the other hand, under stringent PTZ time constraints, *M* coverage cannot locate feasible solutions. In other words, the *M* coverage model cannot adapt to dynamic PTZ time constraints.
(c) The TACZ model can adapt and be scalable to different PTZ time constraints but not the *M* coverage model:
  In Figure 6, Figure 8, Figure 9 and Figure 10, we conclude that the TACZ model can adapt to different values of the PTZ time requirement to identify the sufficiently small number of the deployed sensors needed to cover the network. However, the *M* coverage model cannot adapt to different PTZ time constraints so that it might either deploy too many sensors under loose PTZ time constraints or it cannot locate feasible solutions under stringent PTZ time constraints. For example, in Figure 1c, with the PTZ time constraint *ζ* = 0.5*β*, the PTZ time of the visual sensor *S*_1_ to cover node *C* and the visual sensor *S*_2_ to cover node *C* is smaller than 0.5*β* but is bigger than 0.1*β*. In this case, M2 coverage model can locate the feasible solution when *ζ* = 0.5*β* but the M2 coverage model cannot locate the feasible TACZ model solution when *ζ* = 0.1*β*.

In the fourth set of experiments, we compare the TACZ model with the optimal solutions. Recall that the PTZ WVSN placement problem is proven to be a NP-complete problem [20]. In order to identify the optimal solutions in manageable time, the covered zone is 200 × 200 area with 16 equal sized grids. The possible locations to deploy a visual sensor is at the corners of the grid, so instead of 1000 locations in the earlier experiments, there are 25 possible locations to deploy a visual sensor. We have two important observations:

(a) PTZ capable visual sensors can help to reduce the number of deployed sensors.
  Table 5 shows the benefits of PTZ capability in reducing the number of deployed visual sensors. According to Equation (31), when *ζ* = 0, the TACZ only includes the DCR. It means that the application time requirement is so stringent that the camera is not allowed to PTZ and the optimal solution is the same as the optimal solution without PTZ capability. In this case, the coverage angle of the visual sensor is *θ (*i.e., 90°). On the other hand, when *ζ* = 0.5*β*, the coverage angle of the visual sensor is *θ* + (*β/*2) × 2 (i.e., 180°). As can be observed in the Table 5, the optimal solution for *ζ* = 0.5*β* is almost half the optimal solution for *ζ* = 0. This is because the coverage angle of the visual sensor is double in *ζ* = 0.5*β* as compared to *ζ* = 0.
(b) The proposed algorithm is especially efficient in large scale PTZ WVSN deployment problems.
  When we compare the TACZ model with the optimal solutions in Table 5, we found that only when the focal range is small (*r* = 75), can the TACZ model identify the optimal solutions. This is because the TACZ model iteratively deploys visual sensor at the location that can cover the largest number of uncovered nodes. When the focal range is large, this strategy will leave some small number of unvisited nodes at the later iterations, requiring more visual sensors. However, when the focal range is small, more visual sensor nodes are needed and the TACZ recheck procedure at step 4 can help to remove the deployed sensors whose covered zone is fully covered by sensors at the later stages. In other words, the proposed TACZ model is especially efficient in large scale PTZ WVSN deployment problem.

## 6. Conclusions

By equipping camera sensors with PTZ capability, the coverage area of these visual sensors could be extended. However, PTZ is a mechanical operation that takes longer time (e.g., seconds), and it might not be able to capture the object at the PTZCR in time for real time applications (e.g., intrusion detection). In this paper, we tackle this PTZ time-aware wireless visual sensor network deployment problem. We propose a TACZ model to capture the 2D and 3D coverage constraints (camera focal range coverage, angle zone coverage and camera PTZ adjustment time) in a PTZ WVSN and propose a novel heuristic algorithm, called TADPTZ, to successfully tackle the time-aware visual sensor deployment problem in PTZ WVSNs. From our computational experiments, the solution performance improvement of the TACZ model over the *M* coverage model could be up to 123.2% under loose PTZ time constraints and 55.6% under stringent PTZ time constraints. In addition, the TACZ recheck procedure can help to eliminate redundant visual sensors more efficiently in large PTZ WVSNs. It is concluded that, as compared to the existing *M* coverage model and optimal solutions, the proposed TACZ model could be adaptable and scalable to different PTZ time constraints and large network settings.

## Figures and Tables

**Table 1:** Notations used in the formulation.

Input values:
*L*: the set of possible nodes;
ε : a very small positive value (i.e., ε≅0 and ε>0 );
*r_j_*: sensing range of sensor *j*;
*d_ij_*: Eculidean distance between location *i* and sensor *j*;
$\Psi_{j}\left(D_{j}\right)$ : the deployment cost for placing sensor at location *j*;
*a_ij_*: the relative angle of node *i* to sensor *j* and $0\leq a_{ij}\leq 2\pi$ ;
$\Gamma_{j}\left(a_{ij}\right)$ : the time to adjust the camera at sensor *j* to capture the visual data at node *i* with respect to the relative angle *a_ij_*;
*T*: the maximal allowable time for adjusting the camera on the visual sensor;
*b_j_*: the starting angle of the angle span zone for sensor *j* and $0\leq b_{j}\leq 2\pi$ ;
$\theta_{j}$ : span angle of the DCR for sensor *j* and $0\leq \theta_{j}\leq 2\pi$ ;
$\beta_{j}$ : PTZ angle of the PTZCR for sensor *j* and $0\leq \beta_{j}\leq 2\pi$ ;
Decision variables:
$z_{ij}^{1}$ : =1 if Equation (4) is satisfied; =0, otherwise;
$z_{ij}^{2}$ : =1 if Equation (5) is satisfied; =0, otherwise;
$y_{ij}^{1}$ : =1 if Equation (6) is satisfied; =0, otherwise;
$y_{ij}^{2}$ : =1 if Equation (7) is satisfied; =0, otherwise;
$y_{ij}^{3}$ : =1 if Equation (8) is satisfied; =0, otherwise;
$y_{ij}^{4}$ : =1 if Equation (9) is satisfied; =0, otherwise;
$v_{ij}^{1}$ : =1 if node *i* is covered within the DCR of sensor *j*; = 0, otherwise;
$v_{ij}^{2}$ : =1 if node *i* is covered within the PTZCR of sensor *j*; = 0, otherwise;
*D_j_*: =1 if sensor is deployed at node *j*; = 0, otherwise.

**Table 2:** SR in loose PTZ time constraint.

SR of TACZ Over	Focal Range (*r*)	PTZ Span Angle (*β*)	DCR Span Angle (*θ*)
*M* = 2	41.5%	92.8%	67.4%
*M* = 3	49.0%	123.2%	86.2%

**Table 3:** SR in stringent PTZ time constraint (*ζ* = 0.5*β*).

SR of TACZ Over	Focal Range (*r*)	PTZ Span Angle (*β*)	DCR Span Angle (*θ*)
*M* = 2	17.0%	34.3%	25.7%
*M* = 3	24.0%	55.6%	39.8%

**Table 4:** SR comparisons with respect to *β*.

SR of TACZ Over	*ζ* = 0.3*β*	*ζ* = 0.2*β*	*ζ* = 0.1*β*
*M* = 2	9.5% (75°)	4.8% (20°)	4.2% (10°)
*M* = 3	29.4%	13.8% (75°)	5.4% (40°)

**Table 5:** Optimal solutions comparisons in *θ* = 90°, *β* = 90°.

(Optimal, TACZ)	*r* = 75	*r* = 100	*r* = 125
*ζ* = 0	(16, 16)	(8, 8)	(5, 8)
*ζ* = 0.5*β*	(8, 8)	(4, 6)	(3, 6)
